# Long-Term Health Outcomes of Infantile Spasms Following Prednisolone vs. Adrenocorticotropic Hormone Treatment Characterized Using Phenome-Wide Association Study

**DOI:** 10.3389/fneur.2022.878294

**Published:** 2022-04-13

**Authors:** Monika Baker, Clint C. Mason, Jacob Wilkes, David Sant, Matthew Sweney, Joshua L. Bonkowsky

**Affiliations:** ^1^Division of Pediatric Neurology, Department of Pediatrics, University of Utah School of Medicine, Salt Lake City, UT, United States; ^2^Department of Pediatrics, University of Utah School of Medicine, Salt Lake City, UT, United States; ^3^Intermountain Healthcare, Salt Lake City, UT, United States; ^4^Department of Biomedical Sciences, Noorda College of Osteopathic Medicine, Provo, UT, United States; ^5^Brain and Spine Center, Primary Children's Hospital, Salt Lake City, UT, United States

**Keywords:** pediatric, epilepsy, infantile spasms (IS), ACTH, outcomes-health care

## Abstract

**Objective:**

To determine differences in long-term health and neurological outcomes following infantile spasms (IS) in patients treated with adrenocorticotropic hormone (ACTH) vs. prednisolone/prednisone (PRED).

**Methods:**

A retrospective, case-control study of patients with an International Classification of Diseases, Ninth Revision, Clinical Modifications (ICD-9) diagnosis of IS, identified over a 10-year period from a national administrative database, was conducted. IS patients treated with ACTH or PRED were determined and cohorts established by propensity score matching. Outcomes, defined by hospital discharge ICD codes, were followed for each patient for 5 years. Related ICD codes were analyzed jointly as phenotype codes (phecodes). Analysis of phecodes between cohorts was performed including phenome-wide association analysis.

**Results:**

A total of 5,955 IS patients were identified, and analyses were subsequently performed for 493 propensity score matched patients, each in the ACTH and PRED cohorts. Following Bonferroni correction, no phecode was more common in either cohort (*p* < 0.001). However, assuming an *a priori* difference, one phecode, abnormal findings on study of brain or nervous system (a category of abnormal neurodiagnostic tests), was more common in the PRED cohort (*p* <0.05), and was robust to sensitivity analysis. Variability in outcomes was noted between hospitals.

**Significance:**

We found that long-term outcomes for IS patients following ACTH or PRED treatment were very similar, including for both neurological and non-neurological outcomes. In the PRED-treated cohort there was a higher incidence of abnormal neurodiagnostic tests, assuming an *a priori* statistical model. Future studies can evaluate whether variability in outcomes between hospitals may be affected by post-treatment differences in care models.

## Introduction

Infantile spasms (IS) is a severe pediatric epilepsy disorder typically presenting in the first year of life (1). Hallmarks of IS include spasm-like seizures that occur in clusters with progressive worsening, and a distinctive electroencephalogram (EEG) pattern termed hypsarrhythmia (1, 2). Patients are at risk for adverse long-term outcomes, including increased mortality, risk for intractable epilepsy, and neurodevelopmental impairment (3). Determination of treatment guidelines for IS has evolved over the past several decades (2, 4). First-line treatment for IS consists of steroid or steroid-inducing treatment, but the choice of prednisolone/prednisone (PRED) or adrenocorticotropic hormone (ACTH) has conflicting or equivocal data on efficacy, including time to remission, resolution of hypsarrhythmia, and outcomes (2, 5). Further, ACTH is significantly more expensive without evidence supporting its cost-effectiveness (6). Our goal was to characterize the long-term neurological and other health outcomes for IS, comparing patients who received either ACTH or PRED, using information from a nationwide pediatric clinical administrative database.

## Methods

### Study Design and Participants

We performed a retrospective analysis of data from the Pediatric Hospital Information System (PHIS). PHIS is a nationwide database containing pediatric patient data from 52 children's hospitals (7), including inpatient visit data, as well as some observation, emergency department, ambulatory surgery, and clinic visits data. From PHIS, we identified all patients with an International Classification of Diseases, Ninth Revision, Clinical Modifications (ICD-9) code indicative of infantile spasms (ICD-9-CM: 345.6, 345.60, 345.61), from January 1, 2004 to September 30, 2015. Prior work has indicated the effectiveness of using ICD codes for identifying patients with IS (8). Outcomes for each patient were measured for up to five years after initial IS diagnosis.

### Standard Protocol Approvals, Registrations, and Patient Consents

This project used de-identified data and was not considered human subjects research, and was exempted by the Institutional Review Boards at the University of Utah and Intermountain Healthcare.

### Data Preparation

Data for IS patients was accomplished by identifying patients with an ICD-9-CM code of IS, either 345.60 (Infantile Spasms, without mention of intractable epilepsy) or 345.61 (Infantile spasms, with intractable epilepsy). For inclusion, patients had to receive ACTH or PRED within the 21 days following IS diagnosis. The diagnosis of IS and the administration of ACTH or PRED were determined from PHIS. Patients on both ACTH and PRED (96 patients) or on neither (3,497 patients) were removed. For the remaining 2,362 patients, their ICD-9 codes were converted to phecodes. Phenome-wide association study (PheWAS) is a methodology for evaluating patients by grouped diagnostic codes (9). PheWAS categorizes each ICD-9 code into one of 1866 “phecodes”, which are groups of similar diseases or traits. A phecode was assigned to a patient if they had a matching phecode-associated ICD-9 diagnosis. Phecodes were rounded to the lowest whole integer to reduce granularity in the data for analysis. For instance, the phecode 008.xx indicates an intestinal infection, with the numbers after the decimal point indicating more granular details concerning the phecode (for example, 008.52 indicates an intestinal infection due to *C. difficile*).

The data was first filtered based on the medication given. Patients on ACTH or PRED were retained, patients on neither or both were discarded. For patients with multiple IS diagnoses, the earliest visit was used as the index date. To more accurately ensure that ACTH or PRED was given for an IS diagnosis and not a different diagnosis, additional requirements were as follows: 1. The patient's age at the time of IS diagnosis had to be <1 year (<365 days). 2. The patient's age (in days) at the time of IS diagnosis has to be less than or the same as the age when the patient received the first dose of either ACTH or PRED. 3. The administration of medication needed to occur at no more than 21 days after the initial IS diagnosis.

All diagnoses/ICD codes/phecodes between 6 months and 5 years after initial IS presentation were included for analysis, resulting in 1,916 patients and 66,257 phecodes prior to propensity score matching. Duplicates for phecodes and medical record number were removed. Prior to this removal, data was analyzed for Wilcoxon-Mann-Whitney tests to determine changes in phecode frequencies on the individual level. Chronic condition complex (CCC) data was recorded using two categories: (10) non-neurological CCC codes (termed “Non-Neuro CCC”) and neurological CCC (termed “Neuro CCC”) codes.

CCC determination was used to establish similar patients for matching. For Neuro CCC and Non-Neuro CCC codes, a binary flag (1 yes, 0 no) was used to indicate whether a patient was diagnosed with a chronic condition prior to the initial IS diagnosis. To prevent all patients from receiving a Neuro CCC flag because of their IS diagnosis, and instead only identify those with addition neurological CCCs, the IS CCC flag was removed unless the ICD-9-CM code also indicated a concurrent diagnosis of intractable epilepsy (345.61: Infantile spasms, with intractable epilepsy).

Discharge year was determined based on the first visit for IS to account for changes in IS treatment over time. Sex, race/ethnicity, urban flag, and payer, were all based on a patient's first visit for any diagnosis. Non-Neuro CCC flag and Neuro CCC flag were determined by filtering for phecodes prior to a patient's initial IS diagnosis.

### Propensity Score Matching

Propensity score matching was performed including for sex, payer (government, private, unknown, or other), urban flag (based on Rural-Urban Commuting Area (RUCA) codes), race/ethnicity, year of discharge, Neuro CCC, and Non-Neuro CCC. Propensity score matching was performed in R (version 3.6.1) using the MatchIt package. A 1:1 matching ratio of ACTH to PRED patients was used. Among 1,916 total ACTH or PRED patients, 986 patients matched (493 in each drug cohort). We used k-nearest neighbors for matching and a caliper of 0.20 standard deviations.

To optimize the matching process, the variables discharge year and race were further categorized. Year of discharge was simplified into 3 subgroups: group 1, discharge years 2008–2011; group 2, 2012–2015; and group 3, 2016–2019. Due to the small proportion of persons with Native American, Black Hispanic, and Pacific Island race/ethnicity in our study, these individuals were placed in a single group.

### Statistical Analysis

Frequency calculations of the cohort (ACTH or PRED) phecodes were performed using Python. Differences between the groups were evaluated by either a two-sided Fisher's exact test; or distributional, with the number of distinct occurrences for a given phecode between the two cohorts assessed in rank distribution by the Wilcoxon-Mann-Whitney (WMW) test (SciPy v1.6.1). Although each patient in the ACTH cohort was matched with a patient in the PRED cohort, this similarity was not considered causal enough to justify using paired method tests (11). Phecodes were then sorted by lowest (most significant) *p*-value for each cohort. Percentage differences between the phecodes of the two cohorts were also calculated. Adjusted *p*-values were calculated using a Bonferroni correction. To determine whether there were differences in phecode frequencies at the individual level, as well as between the two drug groups, a Wilcoxon-Mann-Whitney Test was performed on the data prior to duplicate phecode removal.

For sensitivity analysis, to determine whether the hospitals with the most IS patients had a disproportionate effect on the results vs. an overall trend in the data, the holdout (leave one out) method was performed on the five hospitals with the most patients. The top hospital in terms of patient contribution to data was removed prior to matching, and the data analysis was rerun. This was done sequentially for the top five contributing hospitals.

### Data Availability Statement

All data are available on request to the authors. Data preparation was performed in Python (version 3.7.7). Code and software used by the authors are freely available, and if not otherwise indicated, are available at GitHub (https://github.com/Monika-Baker) or upon request.

## Results

**Figure 1:**
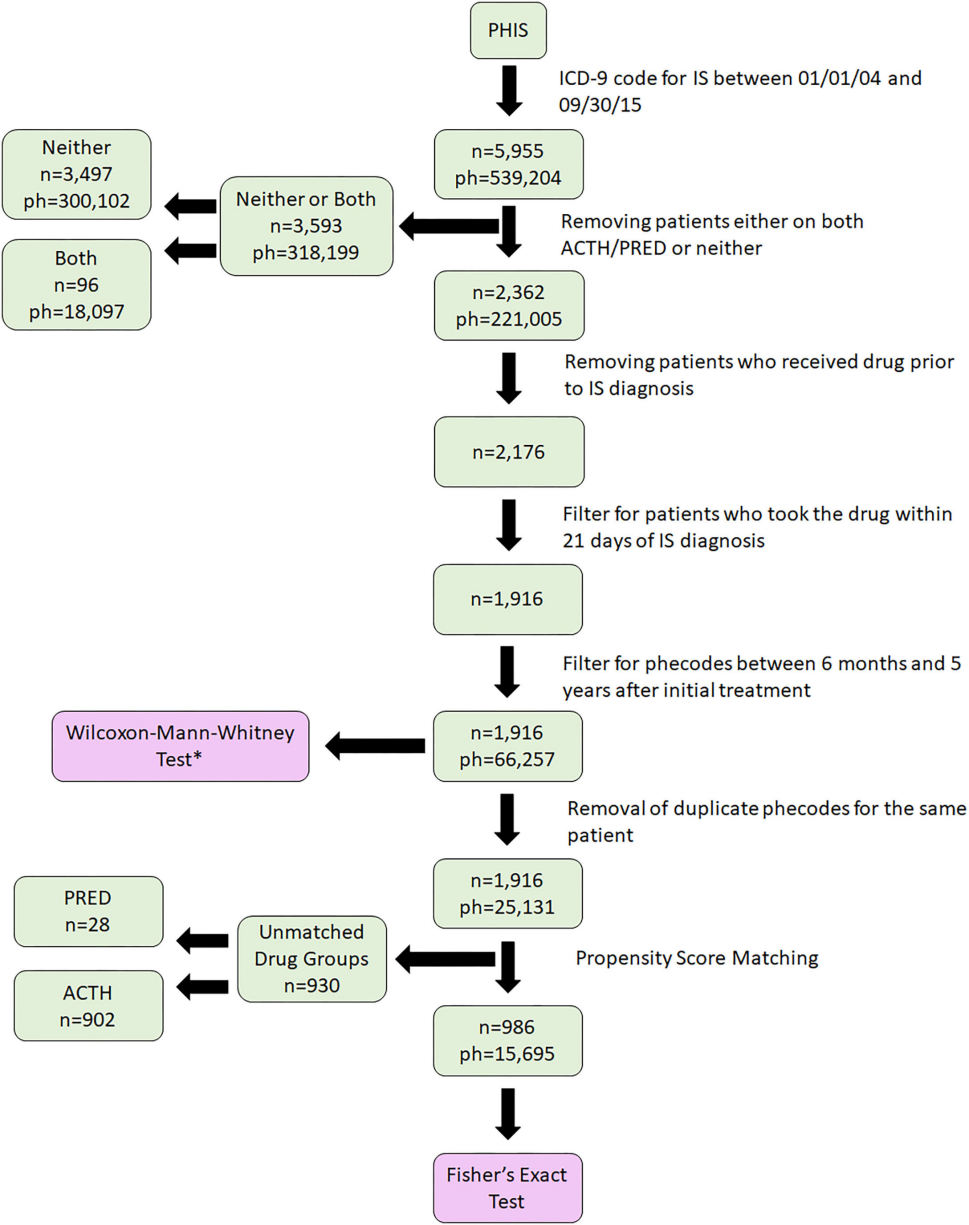
Cohort identification. A total of 5,955 patients with infantile spasms (IS) were identified in Pediatric Hospital Information System (PHIS) using ICD-9 codes. We then filtered for medications and timeframe, performed propensity score matching, and finally, 986 patients were selected for analyses (493 in each drug cohort). IS, Infantile Spasms; ph, phecode; n, number of patients. *The dataset indicated for Wilcoxon-Mann-Whitney test is queried using match results.

**Figure 2:**
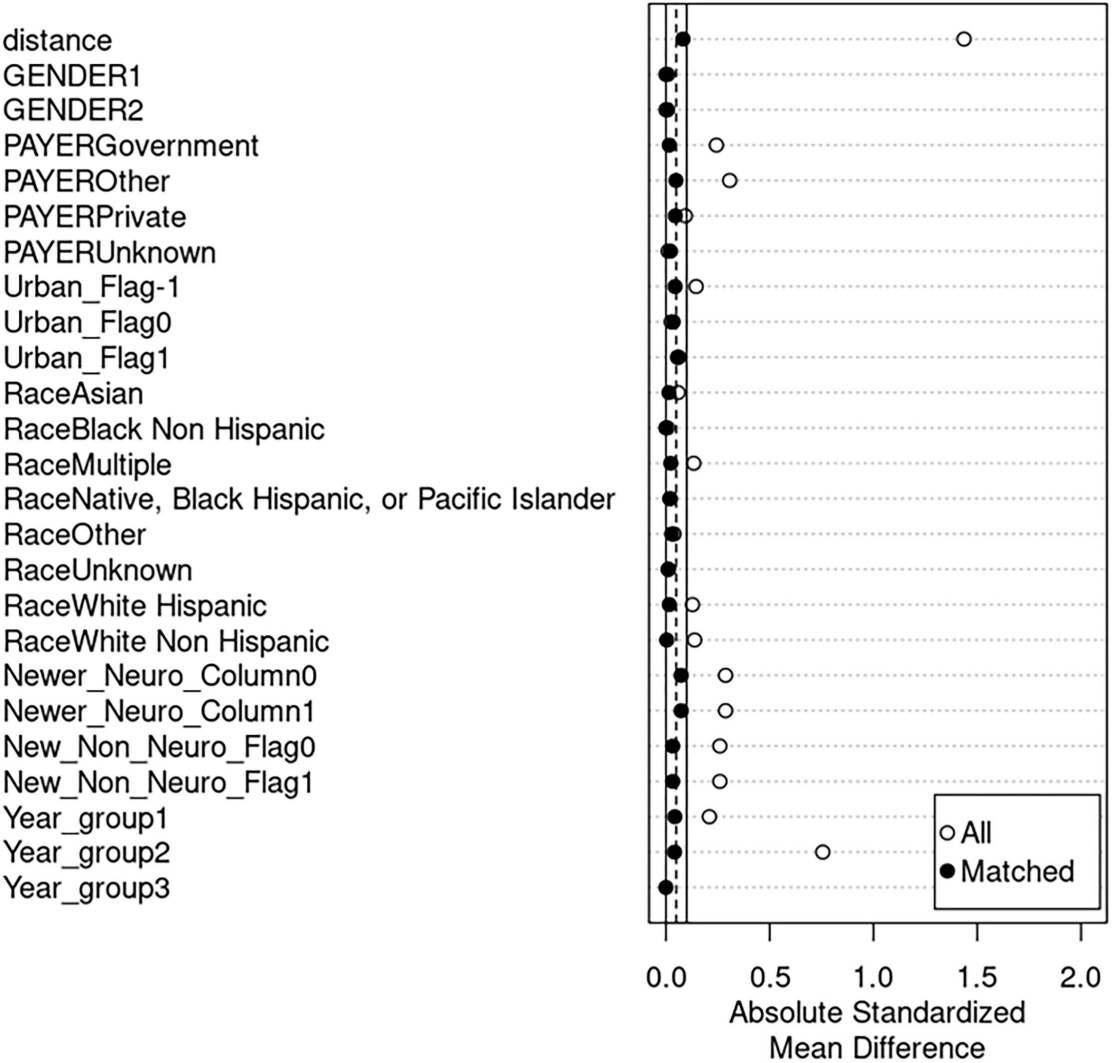
Propensity score matching. Differences in means (y-axis) are shown between the adrenocorticotropic hormone (ACTH) and prednisolone/prednisone (PRED) drug cohorts for covariates before and after matching. Before matching (indicated by white dots), the absolute standardized difference in means has higher values, indicating a higher difference between the cohorts for certain covariates. Following matching (indicated by black dots), the values for the absolute standardized mean difference decrease, indicating a higher degree of similarity between the cohorts when comparing variable frequencies. “Distance” indicates the absolute difference between the propensity scores of matching patients.

From an initial 5,955 IS patients identified, following sorting and exclusions, we identified 1,916 patients who had taken either ACTH or PRED (Figure 1). Selected demographic data are provided in Table 1. After propensity score matching (Figure 2), final cohorts of 493 patients each for ACTH and PRED were established. All ICD-9 codes related to each patient were then collected for 5 years after initial date of IS diagnosis and grouped into phecodes.

**Table 1:** Demographics of the infantile spasms (IS) cohort before and after matching.

	**Prior to matching**		**After matching**	
	**ACTH**	**PRED**	**ACTH**	**PRED**
	***n* (%)**	***n* (%)**	***n* (%)**	***n* (%)**
**Sex**
Female	624 (45%)	233 (45%)	219 (44%)	221 (45%)
Male	771 (55%)	288 (55%)	274 (56%)	272 (55%)
**Race**
White Non-Hispanic	774 (55%)	253 (49%)	248 (50%)	247 (50%)
White Hispanic	164 (12%)	86 (17%)	66 (13%)	69 (14%)
Black Non-Hispanic	168 (12%)	64 (12%)	63 (13%)	63 (13%)
Other	150 (11%)	50 (10%)	51 (10%)	47 (10%)
Multiple	53 (4%)	38 (7%)	34 (7%)	37 (8%)
Unknown	35 (3%)	14 (3%)	15 (3%)	14 (3%)
Asian	38 (3%)	10 (2%)	11 (2%)	10 (2%)
Native, Black Hispanic, or Pacific Islander	13 (1%)	6 (1%)	5 (1%)	6 (1%)
**Insurance**
Government	575 (41%)	278 (53%)	256 (52%)	260 (53%)
Private	607 (44%)	203 (39%)	207 (42%)	196 (40%)
Other	201 (14%)	35 (7%)	26 (5%)	32 (6%)
Unknown	12 (1%)	5 (1%)	4 (1%)	5 (1%)
**Urban flag**
Missing value/other country (−1.0)	31 (2%)	29 (6%)	16 (3%)	21 (4%)
Rural (0.0)	213 (15%)	75 (14%)	68 (14%)	74 (15%)
Urban (1.0)	1,151 (83%)	417 (80%)	409 (83%)	398 (81%)
**Discharge year**
Group 1 (2008–2011)	333 (24%)	176 (34%)	184 (37%)	174 (35%)
Group 2 (2012–2015)	331 (24%)	316 (61%)	280 (57%)	290 (59%)
Group 3 (2016–2019)	731 (52%)	29 (6%)	29 (6%)	29 (6%)
**Chronic neurological conditions**
At least one	427 (31%)	234 (45%)	195 (40%)	213 (43%)
None	968 (69%)	287 (55%)	298 (60%)	280 (57%)
**Non-neurological chronic conditions**
At least one	454 (33%)	237 (45%)	204 (41%)	212 (43%)
None	941 (67%)	284 (55%)	289 (59%)	281 (57%)

**Figure 3:**
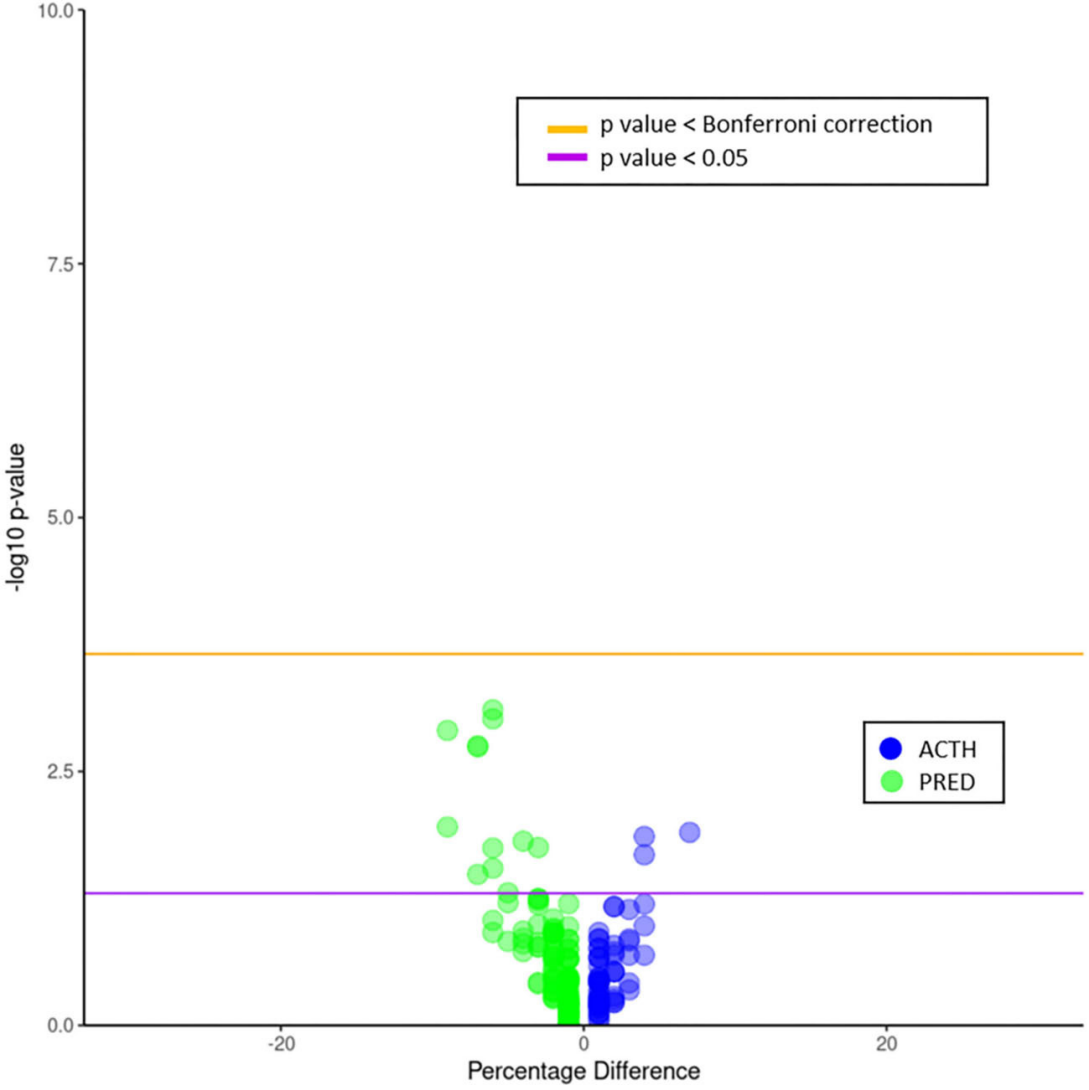
Volcano plot of phecode differences between ACTH and PRED patients. Frequencies are plotted as negative log_10_ of the *p*-value over the percentage difference. The purple line indicates a *p*-value of 0.05; the orange line indicates an adjusted *p*-value using the Bonferroni correction. Neither drug cohort had phecode frequencies above the adjusted *p*-value. However, the PRED cohort had two neurological phecode frequencies above the *p* < 0.05 line, abnormal findings on study of brain/nervous system, and infantile cerebral palsy (percentage differences of 7% for both phecodes). The ACTH cohort also had a neurological phecode frequency above *p* < 0.05 line, hemiplegia (percentage difference of 4%).

Phecode frequencies and percentage differences were compared for ACTH and PRED cohorts (Figure 3). Neither of the treatment groups had phecode frequencies with significant *p*-values following Bonferroni correction. However, the PRED-treated group had two neurological conditions: abnormal findings on study of brain/nervous system, and infantile cerebral palsy with significant *p* < 0.05 values assuming an *a priori* hypothesis of a difference in neurological outcomes (Table 2; percentage rate difference for both was 7%). The ACTH-treated group also had a neurological condition with a frequency greater than *p* < 0.05, hemiplegia (percentage rate difference 4%).

**Table 2:** Unadjusted *p-*values of neurological phecodes, in prednisolone/prednisone (PRED) and adrenocorticotropic hormone (ACTH) cohorts.

**Phecode description**	PRED
Abnormal findings on study of brain and/or nervous system	0.*001812*
Infantile cerebral palsy	0.*032601*
Other conditions of brain	0.148819
Sleep disorders	0.537421
Developmental delays and disorders	0.820108
Epilepsy, recurrent seizures, convulsions	0.917949
**Phecode description**	**ACTH**
Hemiplegia	0.*02090*
Delirium, dementia, and amnestic and other cognitive disorders	0.0677
Strabismus and other disorders of binocular eye movements	0.104934
Hearing loss	0.382294
Neurological disorders	0.556552
Disorders of optic nerve and visual pathways	0.595292

**Figure 4:**
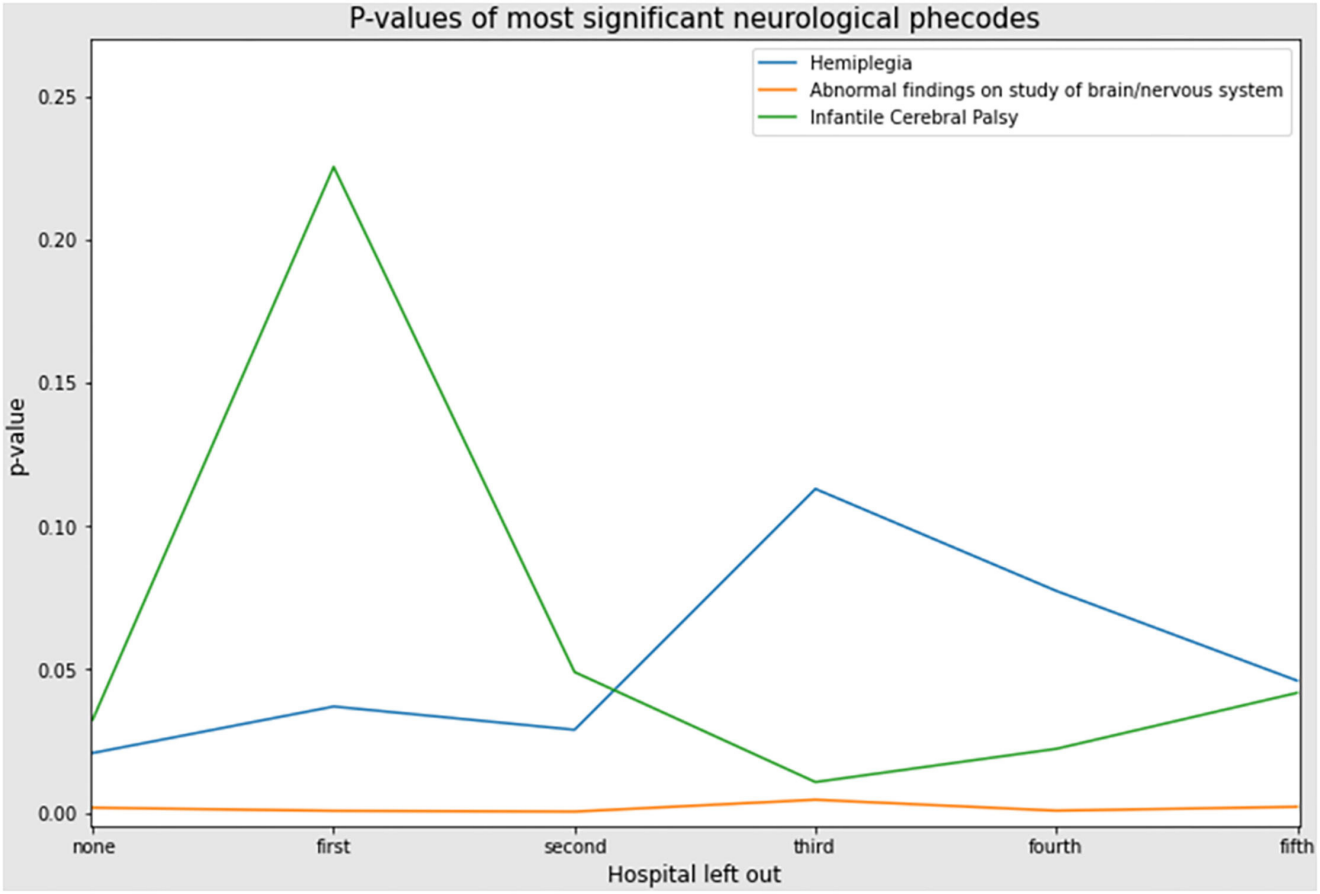
Sensitivity analysis. Graph of leave one out analysis; *p*-value on y-axis and rank order contributing hospitals on x-axis. The top patient contributing hospital was left out prior to propensity score matching and data analysis rerun including Fisher's exact test. This was performed sequentially for each of the top hospitals. Results of *p*-value for the phecode, abnormal findings on study of brain/nervous system, remained similar. However, the *p*-values for infantile cerebral palsy and hemiplegia fluctuated substantially when the first and third hospitals were left out, respectively. This indicates that these hospitals were skewing the frequencies for these two phecodes, and that it is not an overall trend in the data.

Following sensitivity analysis (leave one out/holdout), abnormal findings on study of brain/nervous system retained significance and did not dramatically fluctuate, but hemiplegia and infantile cerebral palsy did fluctuate with *p*-values rising above 0.05 (Figure 4). Analysis with Wilcoxon-Mann-Whitney test indicated that these neurological phecodes were also more prevalent at the individual level (abnormal findings on the study of brain/nervous system and infantile cerebral palsy for PRED patients, hemiplegia for ACTH patients) as well as at the cohort level. However, only abnormal findings on the study of brain/nervous system was robust to the sensitivity analysis.

## Discussion

In this large, national-level, long-term analysis of outcomes for IS patients treated with ACTH or PRED, we found no differences in neurological or non-neurological conditions. However, assuming an *a priori* hypothesis of differences in neurological outcomes and thus not performing Bonferroni multiple comparisons correction, PRED-treated IS patients were more likely, and at higher rates, to have two neurological phecodes, abnormal findings on the study of brain/nervous system and infantile cerebral palsy. ACTH-treated IS patients were more likely, and at higher rates, to have the neurological phecode of hemiplegia, assuming the same *a priori* hypothesis.

Only one neurological finding, abnormal findings on the study of brain/nervous, was robust to sensitivity (holdout) analysis. However, the significance for hemiplegia and infantile cerebral palsy did fluctuate, indicating that the differences in frequencies for these phecodes were driven by data from a single (or few hospitals) hospital. Interestingly, two of the phecodes were more common in the PRED-treated group, following analysis with the Wilcoxon-Mann-Whitney test (“Abnormal findings on study of brain/nervous system” and “Hemiplegia, infantile cerebral palsy”). This indicates that the increases in the frequencies of these two phecodes were driven at the individual level, i.e. multiple instances of the same diagnosis (phecode) in the same patient, from different hospital admissions.

Limitations of the study are its retrospective nature, inherent limitations of matching, and that most of the data from PHIS are from in-patient hospitalization. As such, the in-patient source of the majority of data limits quantification of certain disorders, such as developmental delay. However, although we were unable to quantify the absolute number of IS patients with a diagnosis such as developmental delay, our analysis is still able to evaluate for the ratios or proportions, and thus relative differences, between the PRED and ACTH cohorts. Due to the additional complexities in analysis, for this study, we did not evaluate patients who had taken both PRED and ACTH or other medications (e.g., vigabatrin) (12) An additional limitation of the dataset was that a large number of IS patients were listed with neither ACTH or PRED, suggesting that IS patients treated solely with outpatient prescription management, at least within our inclusion time frame, were not included in our analysis. It is important that our data showed no differences in epilepsy outcomes between the PRED and ACTH groups, which has been a concern regarding IS treatment (2, 4).

It is unclear why there is an observed increase in the PRED group of abnormal findings on the study of brain/nervous system. This phecode encompasses multiple nonspecific neurological findings in various neurodiagnostic tests, including cerebrospinal fluid, radiological tests, and EEGs. Further studies should be performed to identify which of these non-specific findings should be focused on, and whether they are due to differences in side effects or disease management.

In conclusion, we have found that PRED and ACTH treatment for IS have similar long-term outcomes for most health conditions, including for most neurological conditions. Further, our study is one of the few for IS, which considers long-term outcomes including that of non-neurological conditions, while most studies are evaluating immediate treatment response (for example, Grinspan et al.) (13) or evaluating long-term outcomes for only cognitive or epilepsy-related aspects (14, 15). As some outcomes appeared to be correlated with specific hospitals, future studies can evaluate whether variability in outcomes may be affected by post-treatment differences in care models.

## Data Availability Statement

The original contributions presented in the study are included in the article/Supplementary Material, further inquiries can be directed to the corresponding author.

## Ethics Statement

The studies involving human participants were reviewed and approved by University of Utah IRB. Written informed consent from the participants' legal guardian/next of kin was not required to participate in this study in accordance with the national legislation and the institutional requirements.

## Author Contributions

The study was conceived by JB. MB and JW collected data. All authors analyzed data, prepared data for publication, edited drafts of the manuscript, and approved the final manuscript for publication.

## Conflict of Interest

JW was employed by Intermountain Healthcare. The remaining authors declare that the research was conducted in the absence of any commercial or financial relationships that could be construed as a potential conflict of interest.

## Publisher's Note

All claims expressed in this article are solely those of the authors and do not necessarily represent those of their affiliated organizations, or those of the publisher, the editors and the reviewers. Any product that may be evaluated in this article, or claim that may be made by its manufacturer, is not guaranteed or endorsed by the publisher.

## Supplementary Material

Supplementary File 1 Excel file of Fisher's exact test results for phecodes with higher frequencies in the PRED-treated cohort.

Supplementary File 2 Excel file of Fisher's exact test results for phecodes with higher frequencies in the ACTH-treated cohort.

Supplementary Figure 1 Wilcoxon-Mann-Whitney test. To determine if there were differences in frequencies for the same phecode at the patient level, a Wilcoxon-Mann-Whitney test was performed. The phecode file before duplicate removal was used to determine phecode frequencies, and was queried with a patient list after propensity score matching. The results were similar to the results from the Fisher's exact test with abnormal findings on study of brain/nervous system, infantile cerebral palsy, and hemiplegia with *p* < 0.05. However, following Bonferroni correction, there were no significant non-neurological phecodes.
