# Supplementary figures and images for: Long-Term Health Outcomes of Infantile Spasms Following Prednisolone vs. Adrenocorticotropic Hormone Treatment Characterized Using Phenome-Wide Association Study
Source: Front Neurol. 2022 Apr 13;13:878294. doi: 10.3389/fneur.2022.878294 (PMC9043313; doi:10.3389/fneur.2022.878294)

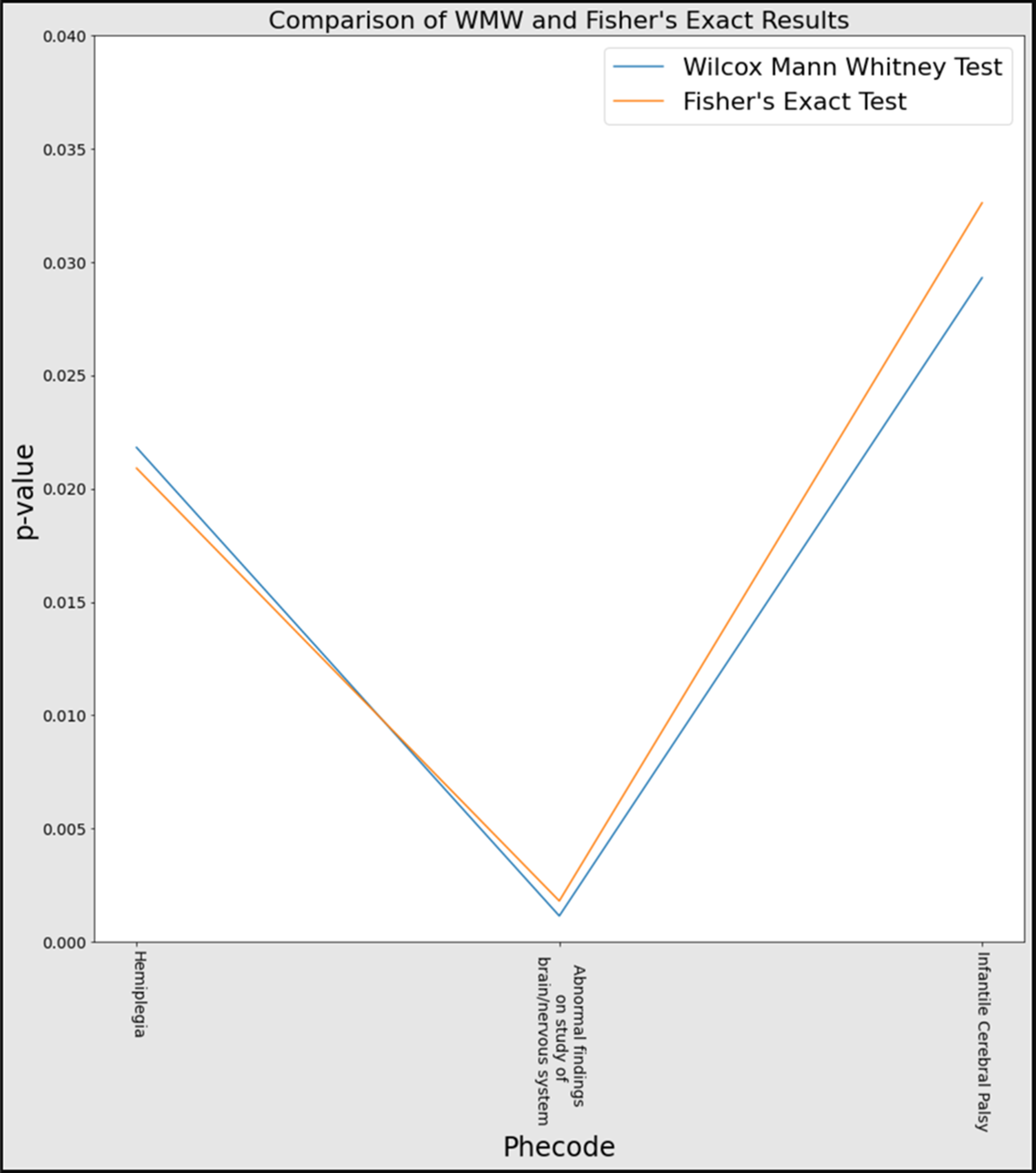

Supplement: Supplementary Figure 1 — Wilcoxon-Mann-Whitney test. To determine if there were differences in frequencies for the same phecode at the patient level, a Wilcoxon-Mann-Whitney test was performed. The phecode file before duplicate removal was used to determine phecode frequencies, and was queried with a patient list after propensity score matching. The results were similar to the results from the Fisher's exact test with abnormal findings on study of brain/nervous system, infantile cerebral palsy, and hemiplegia with p < 0.05. However, following Bonferroni correction, there were no significant non-neurological phecodes. [file Image_1.TIF]
